# Delayed Postoperative Epidural Hematoma Presenting Only with Vesicorectal Disturbance

**DOI:** 10.1155/2013/861961

**Published:** 2013-09-01

**Authors:** Hiroto Kamoda, Tetsuhiro Ishikawa, Masayuki Miyagi, Yawara Eguchi, Sumihisa Orita, Miyako Suzuki, Yoshihiro Sakuma, Yasuhiro Oikawa, Kazuyo Yamauchi, Gen Inoue, Kazuhisa Takahashi, Seiji Ohtori

**Affiliations:** ^1^Department of Orthopaedic Surgery, Graduate School of Medicine, Chiba University, 1-8-1 Inohana, Chuo-ku, Chiba 260-8677, Japan; ^2^Department of Orthopaedic Surgery, Chiba Cancer Center, 666-2 Nitona, Chuo-ku, Chiba 260-8717, Japan; ^3^Department of Orthopaedic Surgery, Kitasato University, School of Medicine, 1-15-1 Kitasato, Minami-ku, Sagamihara, Kanagawa 252-0374, Japan

## Abstract

We present a rare case of delayed onset of epidural hematoma after lumbar surgery whose only presenting symptom was vesicorectal disturbance. A 68-year-old man with degenerative spinal stenosis underwent lumbar decompression and instrumented posterolateral spine fusion. The day after his discharge following an unremarkable postoperative course, he presented to the emergency room complaining of difficulty in urination. An MRI revealed an epidural fluid collection causing compression of the thecal sac. The fluid was evacuated, revealing a postoperative hematoma. After removal of the hematoma, his symptoms disappeared immediately, and his urinary function completely recovered. Most reports have characterized postoperative epidural hematoma as occurring early after operation and accompanied with neurological deficits. But it can happen even two weeks after spinal surgery with no pain. Surgeons thus may need to follow up patients for at least a few weeks because some complications, such as epidural hematomas, could take that long to manifest themselves.

## 1. Introduction

Symptomatic epidural hematoma is a well-known but uncommon postoperative complication of spinal surgery that usually presents with severe back and leg pain. The incidence rate is estimated to be around 0.1% [1, 2]. Most published cases of symptomatic epidural hematoma state that it develops within a few hours after operation. Cabana et al., for instance, reported an average delay of 5.3 hours from the lumbar intervention to the subsequent onset of hematoma symptoms [3].

Even rarer still are cases of delayed symptomatic epidural hematoma. Uribe et al. [4], who defined “delayed” as an occurrence more than three days after operation, reported seven cases in his study. Sokolowski et al. reported four cases of delayed symptomatic epidural hematoma without coagulopathy [5]. In these cases, though, the initial symptoms included severe pain and muscle weakness at the level of previous surgery, the same symptom pattern that accompanies hematomas occurring shortly after surgery.

 We now report a case of postoperative epidural hematoma developing two weeks after surgery whose only presenting symptom was vesicorectal disturbance.

## 2. Case Report

**Figure 1:**
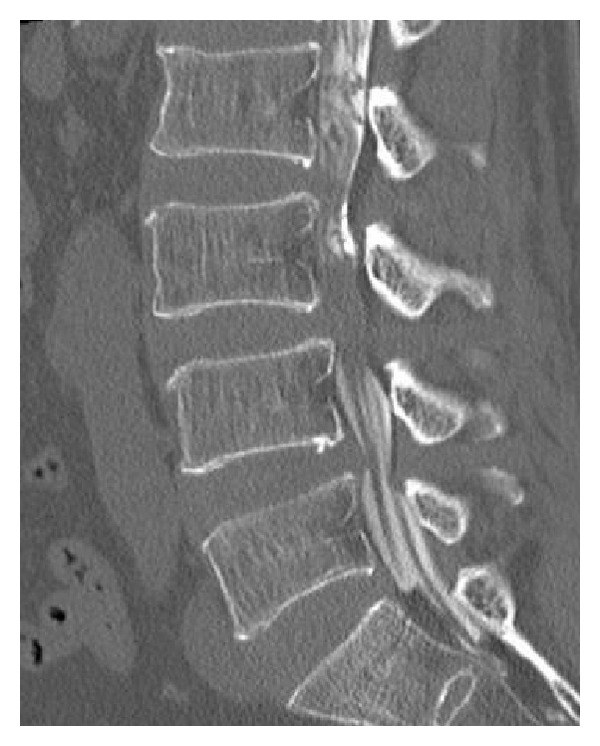
Preoperative imaging. Sagittal reconstruction of a postmyelography computed tomography (CT) scan demonstrates L2-5 multiple spinal stenosis and concurrent grade I degenerative spondylolisthesis at L3/4 and L4/5.

A 68-year-old man with a medical history of hypertension was referred to our hospital with progressively increasing lower back and left leg pain despite analgesic treatment. Preoperative imaging revealed degenerative spinal stenosis from L2 to L5 with spondylolisthesis at L3 to L4 and L4 to L5 (Figure 1). Preoperative neurological examination detected no motor or sensory deficits. The patient did not report any bowel or bladder function loss at that time. He also had no history of prior anticoagulation therapy, and preoperative coagulation studies were normal.

The patient underwent L2 to L5 decompressive laminectomies and L3 to L5 instrumented posterolateral spinal fusion with autograft bone. No dural tears occurred intraoperatively. After achievement of excellent hemostasis, a surgical drain was placed at the time of closure. Intraoperative blood loss was approximately 500 mL.

On postoperative day 2, the drain was removed, and progressive physical therapy was started. His postoperative course was unremarkable. His symptoms resolved, and he was discharged on day 14. That same evening he felt a sensation of lower abdominal bloating.

**Figure 2:**
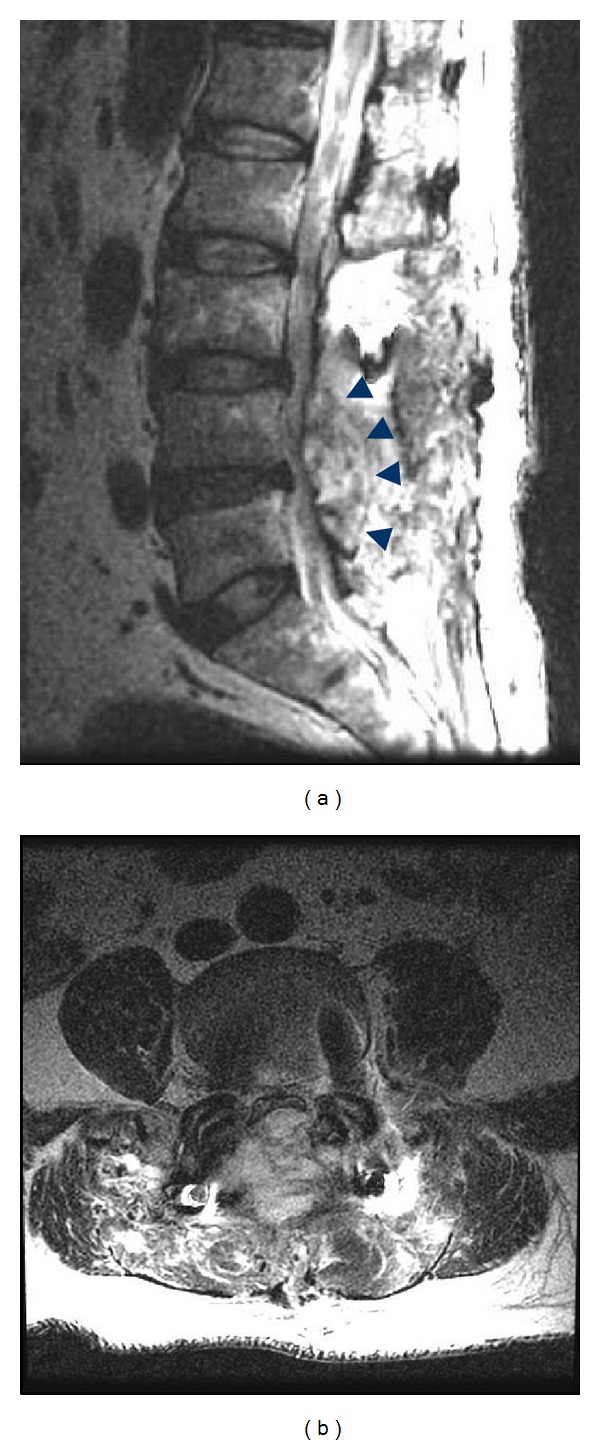
(a) Magnetic resonance imaging (MRI) T2-weighed sagittal image acquired 15 days postoperatively demonstrates a convex-shaped lesion at the surgical site (▲) compressing the thecal sac. (b) MRI T2-weighed axial image shows a posterior high intensity area completely flattening the thecal sac from both posterior right and posterior left sides.

**Figure 3:**
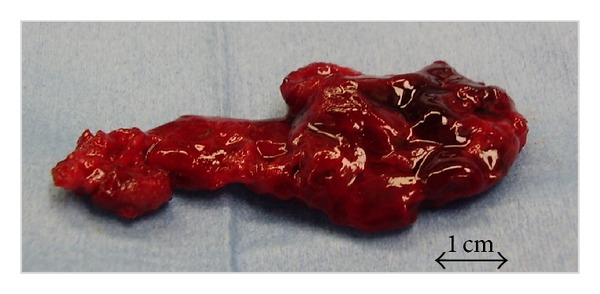
A large consolidated hematoma (4.5 cm × 2 cm) was removed.

**Figure 4:**
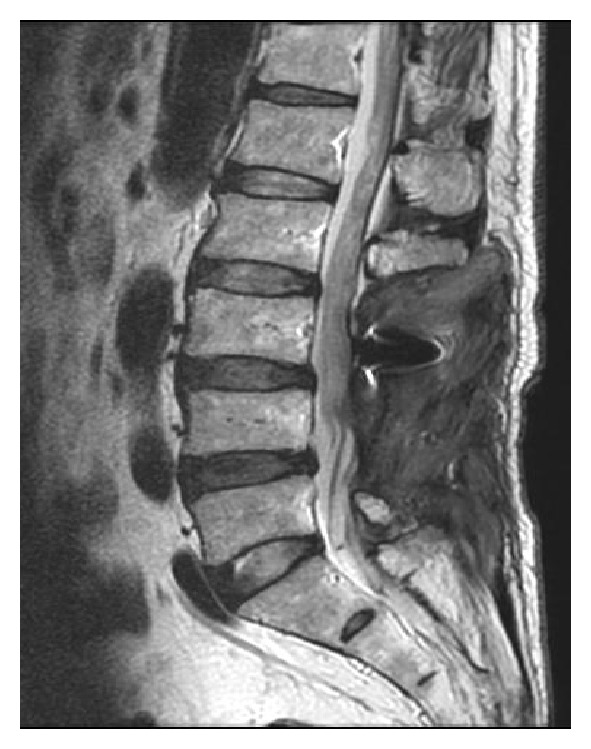
MRI T2-weighed sagittal image after hematoma evacuation shows no compression of the thecal sac.

The next morning (postoperative day 15) he presented to the emergency room complaining of difficulty in urinating. His bladder was found to contain approximately 500 mL of urine. He did not report any leg or back pain. Physical examination revealed no motor or sensory deficits. Emergent lumbar magnetic resonance imaging (MRI) visualized a T2 high weighed epidural mass at the surgical site flattening the thecal sac from L4 to L5 (Figure 2). We immediately performed surgical evacuation manually with irrigation and in the process discovered a large consolidated hematoma (4.5 cm × 2 cm), which we removed (Figure 3). No obvious source of bleeding could be identified. We placed an epidural drain in the surgical site before closure, which we kept there for 5 days. Postoperative MRI showed complete resolution of the epidural hematoma (Figure 4). After evacuation, his symptoms immediately disappeared, and his urinary function recovered completely. We allowed him to walk again after the removal of drain, and he was discharged home on day 10.

## 3. Discussion 

Several published studies have discussed the etiology of postoperative epidural hematoma. Kou et al. [2] identified multilevel procedures and the presence of preoperative coagulopathy as possible significant risk factors. Awad et al. [6] divided potential risk factors into two categories, preoperative and intraoperative factors. Significant preoperative risk factors included nonsteroidal anti-inflammatory use and patient age more than 60 years; significant intraoperative risk factors included multiple-level operation, anemia, and large blood loss. Sokolowski et al. [7] reported that age greater than 60 years, multilevel procedures, and preoperative international normalized ratio (INR) correlated with postoperative hematoma volumes. Even though our patient indeed had several recognized risk factors—age more than 60 years, use of analgesic agents, and multilevel surgery—he had no neurological symptoms during his hospitalization. This would imply the nonexistence of an epidural hematoma during the early postoperative period.

Regarding delayed postoperative epidural hematoma, Parthibian and Majeed described one such case which developed following an episode of violent twisting movement [8]. Since our patient had no symptoms at his time of discharge on postoperative day 14 but then had developed enuresis by the next morning without any episode of violent movement, we would suspect that a general increase in activity after discharge triggered epidural bleeding and a growing hematoma.

The other unusual aspect of our case is that our patient only complained of difficulty urinating and did not report pain or muscle weakness at the time of onset. Symptomatic hematoma usually starts with a stabbing pain at the operative site, followed by paresthesia, radicular pain, and neurologic palsy [9]. In our case, the MRI showed the hematoma at the L4-5 level pressing the thecal sac from the posterior side. While our literature search discovered several reports of postoperative epidural hematoma causing cauda equina syndrome [10–14], we did not find any reports of hematoma causing only vesicorectal disturbance without severe pain or muscular weakness. Because urinary function innervation is from S2 to S4, dysuria theoretically could occur if only the central part of thecal sac was being compressed at the L4-5 level. However, since central compression was not the type of thecal sac compression in our patient, the reason for presenting only with vesicorectal disturbance remains unknown and further study is thought to be needed on this point.

Although most surgeons will leave in drains at the surgical site in an effort to prevent the formation of a hematoma following lumbar spine surgery, controversy persists as to whether indwelling drains are effective in preventing hematomas, especially as several studies have claimed that surgical drains are not always necessary for lumbar spine surgery. Brown and Brookfield [15], for instance, performed a randomized study comparing lumbar spine surgery patients who had a drain placed before closure with those who did not, and they found that no patient in either group developed a hematoma. Similarly, Kanayama et al. [16] reported that use of a drain had no influence on the risk of hematoma in single level lumbar decompression. In our case, a symptomatic hematoma formed despite our placing a surgical drain, indicating that surgical drains may not be sufficient for preventing hematoma-related complications.

Patients with symptomatic hematomas are often treated with surgical evacuation immediately to prevent neurological sequelae. Lawton et al. [1] recommended immediate surgical evacuation of the hematoma based on their study findings that patients taken to surgery within 12 hours had better neurological outcomes. In our case, being able to perform surgical evacuation approximately five hours after our patient presented to the emergency room may have been a major factor in his recovering completely.

In conclusion, delayed postoperative epidural hematoma is a rather rare complication of spinal surgery. When this complication arises, it usually presents with severe leg or back pain, but not always, as our patient demonstrated. This case illustrates the need for surgeons to follow up patients for at least a few weeks after lumbar spine surgery because some complications, such as epidural hematomas, could take that long to manifest themselves.
